# Intraoperative Parathyroid Hormone Monitoring Corroborates the Success of Parathyroidectomy in Children

**DOI:** 10.4274/jcrpe.1401

**Published:** 2014-09-05

**Authors:** Ahmet Çelik, Emre Divarcı, Zafer Dökümcü, Orkan Ergün, Samim Özen, Damla Gökşen, Şükran Darcan, Yeşim Ertan

**Affiliations:** 1 Ege University Faculty of Medicine, Department of Pediatric Surgery, İzmir, Turkey; 2 Ege University Faculty of Medicine, Department of Pediatric Endocrinology, İzmir, Turkey; 3 Ege University Faculty of Medicine, Department of Pathology, İzmir, Turkey

**Keywords:** parathyroid hormone, hyperparathyroidism, Parathyroid adenoma, parathyroidectomy

## Abstract

**Ob­jec­ti­ve:** To assess the efficacy of intraoperative parathyroid hormone (PTH) monitoring in evaluating the outcome of parathyroidectomy in pediatric patients.

**Methods:** Intraoperative PTH monitoring during parathyroidectomy was performed in five children (3M, 2F); three had parathyroid adenomas (single gland disease) and two had primary hyperplasia. One patient had undergone two previous surgical interventions to remove the parathyroid glands, but the PTH levels had remained high with persistence of symptoms. Immunoradiometric analysis was used for PTH measurements. Preoperative PTH values were obtained to monitor the baseline levels. Serum samples were collected 20 minutes after removal of the adenoma/parathyroid gland(s) and PTH levels were compared with preoperative values. Specimens were also confirmed by frozen sectional examination.

**Results:** Mean age of the patients was 11 years (range: 3 months-16 years). Mean preoperative PTH values were 633.3±579 pg/mL (range: 143-1300

pg/mL). Intraoperative values decreased to 18.7±5.5 pg/mL (range: 8-27 pg/mL) following removal of the gland(s). Normal calcium levels were achieved with adequate management following surgery. One patient (with multiple surgeries and found to have an ectopic parathyroid gland) had hungry bone syndrome after the operation and was treated successfully. There were no major complications. All patients maintained normal calcium/phosphorus levels in the follow-up period, ranging from 2 to 5 years.

**Conclusion:** An ectopic parathyroid gland or another undetected adenoma can be overlooked during surgery. Owing to the short life of the hormone, intraoperative PTH monitoring to determine PTH clearance proved to be a feasible marker for adequacy and safety of surgery and “cure”.

## INTRODUCTION

Primary hyperparathyroidism (PHPT) is characterized by overproduction of parathyroid hormone (PTH) from abnormal parathyroid gland(s). This is a very rare disorder in childhood - approximately 1%-2% of all patients with PHPT are children. Deleterious effects of PHPT can be seen especially in the urinary system and bone tissue (1). Removal of abnormal functioning parathyroid gland(s) is the main objective in the treatment of PHPT. Surgical excision should be performed by conventional technique as bilateral neck exploration or minimally invasive parathyroidectomy (2,3,4,5). The removal of all abnormal functioning parathyroid tissue is necessary to achieve a satisfactory result. 

In recent years, PTH monitoring during parathyroidectomy became more popular in adults to improve cure rates (4,6,7,8). There are very few reports about PTH monitoring in childhood (5). In this study, we aimed to report the efficacy of intraoperative PTH monitoring as a useful adjunct to corroborate the success of parathyroidectomy in children with PHPT.

## METHODS

Five pediatric patients (3M, 2F) with PHPT were treated surgically in our institution between 2006 and 2010. The diagnosis was confirmed by laboratory tests [calcium (Ca), PTH levels] and imaging studies in patients with the clinical signs of hypercalcemia. Patients were examined by neck ultrasonography (USG) and MIBI-scintigraphy in routine preoperative work-up. Computerized tomography (CT) or magnetic resonance imaging (MRI) were also used if the localization of the lesion was uncertain. Patients underwent medical therapy with 4 diuretic and bisphosphonate before surgery. 

Intraoperative PTH monitoring is used as a standard procedure during parathyroidectomy. PTH levels are measured by immunoradiometric analysis before and during surgery. Normal PTH level reference was accepted as 10-65 pg/mL. Baseline PTH levels were obtained from all patients preoperatively and 20 minutes after resection of the lesion, serum PTH levels were checked again. Excised tissue underwent frozen sectional examination simultaneously during the surgery. 

The surgical approach consisted of limited neck exploration with single-gland parathyroidectomy in patients with isolated adenoma as assessed by preoperative work-up studies and total parathyroidectomy with autotransplantation for patients with multiple gland disease or hyperplasia. In our study, a curative resection was defined as more than a 90% decrease in PTH levels at 20 minutes following the resection, compared to baseline (9). If PTH levels failed to decrease, exploration for an additional hyper-functioning parathyroid tissue was deemed necessary. After resection of the additional lesion, PTH levels were checked again. This procedure was continued until achieving more than 90% decrease in PTH levels. Serum Ca and PTH levels were followed regularly after surgery. The data were reported as mean ± standard error of the mean.

## RESULTS

The patients’ mean age was 11 years and ranged from age 3 months to 16 years. Major presenting symptoms were listed as headache, abdominal pain, feeding intolerance, mental retardation and walking difficulty (Table 1). Only one patient had a palpable nodule on physical examination. The other patients had no physical signs. None of the patients had a family history of parathyroid disease.

Hyperparathyroidism was diagnosed by laboratory tests as hypercalcemia and increased levels of PTH. Detailed neck USG demonstrated parathyroid adenomas in three patients. Patient 3 had primary hyperplasia, but no lesion could be demonstrated on imaging studies preoperatively. In this patient, total parathyroidectomy was performed by bilateral exploration and half of the one gland was grafted to the left forearm. A significant decrease of PTH level occurred following the total excision. Patient 4 had persistent hypercalcemia, but no pathological lesions were demonstrated by radiological investigations. This patient had primary hyperplasia and had undergone two previous insufficient parathyroidectomies in our clinic previously. At the final operation, she underwent total thyroidectomy for the possibility of ectopic localization, but PTH levels failed to decrease. Persistent exploration for an ectopic parathyroid tissue became necessary and finally, an ectopic tissue was found close to the common carotid artery. This lesion was confirmed to be parathyroid tissue by frozen section examination and more than 90% decrease in PTH levels was achieved ultimately. A normal Ca level was achieved by medical replacement therapy in this patient. 

**Figure 1:**
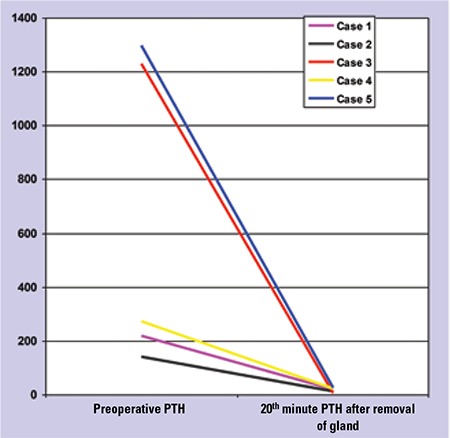
The significant decrease in intraoperative PTH levels after removal of the pathological parathyroid tissues

The clinical disorders leading to hyperparathyroidism were solitary adenomas in three patients and primary hyperplasia in two patients. After removal of the lesions, preoperative PTH levels decreased significantly from 633.3±579 pg/mL to 8.7±5.5 pg/mL (>90%) (Figure 1). Similarly, preoperative Ca levels decreased to normal levels after surgery. 

No major complications occurred after surgery. Only one patient with multiple surgeries had hungry bone syndrome after the operation but was treated successfully with medical treatment. Patient 3 with primary hyperplasia necessitated continuous oral Ca supplementation postoperatively. All patients had normal Ca and PTH levels at postoperative follow-up (2-5 years).

## DISCUSSION

PHPT is defined as excessive secretion of PTH from the parathyroid gland(s). It is characterized by hypercalcemia, increased levels of PTH and slight hypophosphatemia (10). Bone diseases and nephrolithiasis are the major clinical conditions caused by PHPT. Peptic ulcer, hypertension, weakness, weight loss, anorexia, constipation, vomiting, pathological bone fractures, hematuria and polyuria can also develop frequently in these patients.

PHPT and its diagnosis in children is quite rare (11).The largest series to date limited to strictly pediatric patients (5-19 years old) comprised 52 patients, encountered in the course of a 30-year period (12). 

Disorders that can cause PHPT in adulthood are listed as solitary adenomas (90%), primary hyperplasia (9%-10%), and parathyroid carcinomas (0.5%-1%) (13,14). In children, the majority of the lesions (65%) are solitary adenomas, followed by hyperplasia (27%) (12). Several genetic diseases can be seen in children with hyperparathyroidism. Familial hypocalciuric hypercalcemia is caused by the mutation of the Ca-sensitive receptor gene. In homozygous cases, the patients present with excessive hypercalcemia and increased PTH levels in the neonatal period (15,16,17). The heterozygous cases are generally asymptomatic and diagnosed incidentally at any age (17). MEN1, MEN2A and familial hyperparathyroidism are other inherited diseases which can present with hyperparathyroidism in childhood.

Operative treatment for PHPT in children is individualized but can vary from single-gland parathyroidectomy for isolated adenoma to subtotal or total parathyroidectomy with autotransplantation for hyperplasia. The overall success rate of surgical treatment at PHPT in pediatric patients is excellent. The use of preoperative localization imaging with sestamibi scintigraphy or high-resolution USG coupled with intraoperative PTH assay has made more minimally invasive unilateral image-guided explorations possible in highly selected patients (12). In appropriate patients, this has been our preferred approach. However, traditional bilateral neck exploration remains the standard approach, especially for patients with suspected multiglandular disease.

The main objective in the treatment of PHPT is to remove all of the hyper-functioning parathyroid tissues. This can be accomplished by conventional bilateral neck exploration or minimally invasive technique. In adulthood, a 94% cure rate was achieved by bilateral neck exploration (18). There are very few reports on results of surgical treatment in children with PHPT due to the rarity of this condition in the pediatric population (Table 2) (1,5,12). 

Several options have been reported for timing of PTH testing during surgery (7,8). Many surgeons accept a successful resection as more than a 50% decrease at the 10th minute, but this could be misleading in multi-glandular disease (6,7,8). In our study, a curative resection is defined as a more than 90% decrease in PTH levels from baseline at 20 minutes following resection (9). If PTH levels failed to decrease, exploration for an additional hyper-functioning parathyroid tissue was deemed necessary. After resection of the additional lesion, PTH levels were checked again and the procedure continued until achievement of a more than 90% decrease in PTH levels.

Libansky et al (1) performed bilateral neck exploration for treatment of PHPT in 10 children. These authors did not use intraoperative PTH monitoring during surgery. Nine of these patients were treated successfully by one single operation, but one patient necessitated reoperation due to persistent hyperparathyroidism. In the second operation, they performed intraoperative PTH testing and found an ectopic parathyroid tissue within the carotid sheath, a finding similar to one of the patients in our series. 

Minimally invasive parathyroidectomy (MIP) was performed in 12 children by Durkin et al (5). This team preferred to perform MIP in patients with lesions which were localized by preoperative imaging studies (USG or Technetium- 99m sestamibi scanning). Eleven of the 12 patients were treated by MIP successfully (11/12, or 89%). One patient required conversion to bilateral neck exploration due to inadequate decrease in PTH levels (<50% in 15th minute). 

In our study, three children with solitary adenoma underwent MIP. Two patients with primary hyperplasia necessitated total parathyroidectomy with autotransplantation by bilateral neck exploration. Patient 4 had two previous unsuccessful parathyroidectomies, but the hyperparathyroidism persisted. At the third operation, due to the possibility of an ectopic localization, a total thyroidectomy was performed, but PTH measurements failed to decrease intraoperatively. So, we continued to explore for an ectopic parathyroid tissue and ultimately found an ectopic tissue situated close to the common carotid artery. 

In conclusion, our results show that an effective treatment was achieved in all our patients by intraoperative PTH monitoring, with assistance of frozen-section pathological evaluation during parathyroidectomy. Thus, despite the low number of patients in this series, we would recommend intraoperative PTH monitoring to corroborate the success of parathyroidectomy in children.

## Figures and Tables

**Table 1:**
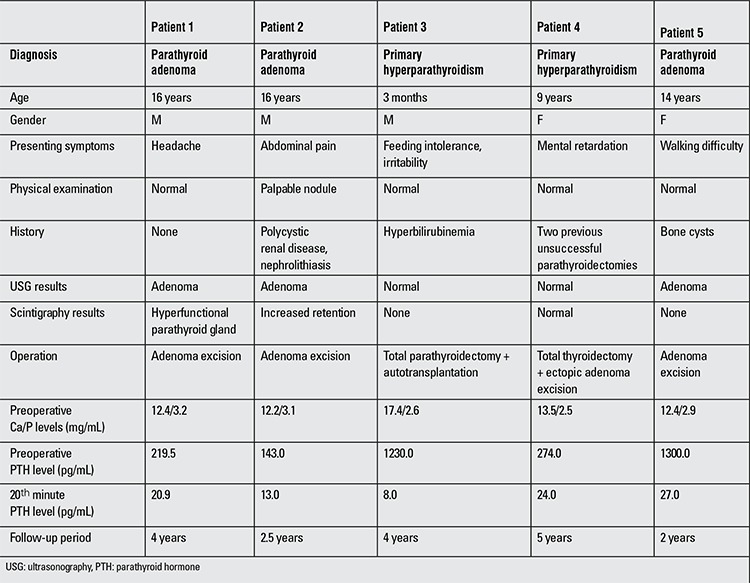
The demographic data of the patients

**Table 2:**
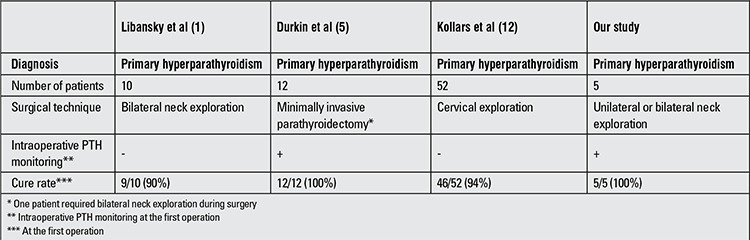
A summary of results of recent studies on parathyroidectomy in children (<18 yrs)
